# Nursing Students’ Perceptions of Barriers, Facilitators and Solutions in Their Role as Health Promoters: Findings from a Qualitative Study

**DOI:** 10.3390/nursrep15070232

**Published:** 2025-06-25

**Authors:** Gloria Modena, Beatrice Mazzoleni, Anna Sponton, Orejeta Diamanti, Giovanna Artioli, Gaia Monti, Valentina Negri, Federica Dellafiore

**Affiliations:** 1 Department of Biomedicine and Prevention, University of Tor Vergata, 00133 Rome, Italy; maria.modena@humanitas.it (G.M.); orejeta.diamanti@iov.veneto.it (O.D.); 2 IRCCS Humanitas Research Hospital, 20089 Rozzano, Italy; anna.sponton@humanitas.it; 3 Department of Biomedical Sciences, Humanitas University, 20072 Milan, Italy; beatrice.mazzoleni@humanitas.it (B.M.); valentina.negri@st.hunimed.eu (V.N.); 4 Department of Medicine and Surgery, University of Parma, 43125 Milan, Italy; giovanna.artioli@unipr.it; 5 Division of Otolaryngology and Head and Neck Surgery, European Institute of Oncology, 20141 Milan, Italy; gaia.monti@ieo.it; 6 Department of Life Science, Health, and Health Professions, Link Campus University, 00165 Rome, Italy

**Keywords:** health promotion, nursing students, education, clinical practice, qualitative research

## Abstract

**Background**: Nurses play a crucial role in health promotion (HP) policy and in encouraging healthy behaviors. However, challenges persist in effectively integrating HP as a core component of healthcare systems and nursing practice. Therefore, it is essential to develop specialized and advanced competencies in nursing students, incorporate HP into nursing academic curricula, and better understand the factors influencing nursing students’ development of these competencies—an area that remains underexplored. Accordingly, this study explores and describes the factors nursing students perceive as essential in developing competencies in HP and in fulfilling their role as health promoters. **Methods**: A qualitative study was conducted using Reflexive Thematic Analysis. Data were collected through semi-structured interviews with 19 nursing students. **Results**: Five main themes emerged: (1) Training Needs of Future Health Promoters, (2) Navigating Time Constraints in Health Promotion, (3) Nurses’ Awareness of Their Role in HP, (4) Perceived Need to Adopt Healthier Lifestyles, and (5) Challenges of Lifestyle Management. Key facilitators included strong mentorship and well-structured educational programs; barriers included insufficient clinical exposure and time constraints. Additionally, students emphasized the need for HP to be a core aspect of nursing curricula. While acknowledging existing obstacles, participants also identified practical solutions that could be integrated into training and practice to enhance the impact of HP in nursing care. **Conclusions**: The study highlights significant gaps in HP education for nursing students and the need for curriculum reforms to better prepare them as health promoters. Future research should consider the perspectives of clinical mentors and evaluate the effectiveness of innovative teaching methods—such as simulation-based training—in strengthening students’ HP competencies.

## 1. Background

Health promotion (HP), codified for the first time in 1986 by the World Health Organization (WHO) through the Ottawa Charter, is defined as “the process of enabling people to increase control over, and to improve their health” [1]. This marked a milestone in global health policies, emphasizing health as a resource to be preserved and nurtured in everyday life. Today, the principles of the Ottawa Charter remain highly relevant. As highlighted during the 9th Global Conference on HP in Shanghai (2016), HP is more essential than ever in addressing today’s interconnected challenges and fulfilling the promise of sustainable development [2]. Globalization, the internet, and climate change have radically transformed the world, introducing unprecedented threats and challenges to human health and well-being, and marking a new and complex frontier for HP [3].

One of the most pressing issues HP must address is the rising prevalence of chronic diseases, the leading causes of death and disability in high-income countries. Globally, over 70% of deaths are attributed to chronic diseases, with annual treatment costs estimated at USD 4.5 trillion, a figure expected to rise by 2030 [3]. Most of these diseases stem from four modifiable and unhealthy behaviors: smoking, poor nutrition, physical inactivity, and excessive alcohol consumption. These behaviors are closely linked to disability and premature mortality. Consequently, global HP policies are urgently needed to support individuals in modifying these behaviors, thereby reducing the incidence of illness, suffering, and early death [2].

In this context, nurses—key healthcare professionals [4]—play a vital role in empowering individuals, families, and communities to take greater control over their health and well-being [5]. Nurses are uniquely positioned for this role due to their frequent and direct contact with patients, positive attitudes, and a strong commitment to HP [6]. Their effectiveness is also supported by their professional knowledge, skills, and philosophy [7]. Through HP strategies, nurses contribute to reducing health disparities by addressing the upstream determinants of chronic diseases associated with an unhealthy lifestyle [8]. Specifically, they are instrumental in promoting treatment adherence [9], improving quality of life [5], and providing overall patient support and empowerment [10].

HP is therefore considered a fundamental responsibility of every nurse [11]. However, effectively integrating HP as a core component of healthcare systems and nursing practice remains a challenge [3,12]. Several personal and systemic barriers hinder the delivery of HP in nursing practice [13], with role confusion among nurses being a significant issue [14]. Recent studies on nursing practice in HP reveal fragmented evidence, and there is still no consensus on the scope and boundaries of nurses’ roles in this field [15]. Moreover, health system planners and managers often provide vague policy guidelines on HP, further contributing to this role ambiguity and potentially undermining the success of HP programs [16].

A critical strategy to overcome these challenges is the developing advanced competencies among nursing students [17], incorporating HP into nursing academic curricula [12,18,19]. The preparation of nurses for the health promoter role must begin during academic training [19,20]. Preparation for HP is multifaceted, requiring a multidisciplinary approach that integrates knowledge and skills across various fields. HP in nursing extends beyond disease prevention and behavior change to fostering a culture of health that encompasses specific knowledge, attitudes, values, and skills [5,18]. Thus, nursing education must emphasize HP significantly to ensure graduates are equipped to apply these concepts effectively in clinical practice [21].

Over the past three decades, a growing body of research has examined nursing students’ preparation for and attitudes toward HP in both European and non-European contexts. While these studies have contributed valuable insights, they also consistently point to a fragmented integration of theory and clinical practice, which hinders the development of a holistic understanding of HP among nursing students [20]. Despite the increasing attention paid to the topic, most existing studies have adopted a predominantly descriptive approach and have not extensively explored the subjective experiences of nursing students. Little is known about the factors that, from the students’ own perspectives, support or hinder the acquisition of HP competencies and the development of their role as health promoters. This represents a critical and underexplored gap in current knowledge, especially considering the growing complexity of health systems and the need for nurses to be adequately prepared for HP roles.

To address this gap, the present study adopts a qualitative and student-centered approach to explore the perspectives of nursing students regarding the factors they consider essential in developing their competencies in HP and in shaping their role as health promoters. By gaining insight into students’ lived experiences, values, and interpretations, this study aims to inform the design of educational strategies and curricular innovations that can better align nursing education with the demands of contemporary health systems.

## 2. Materials and Methods

### 2.1. Study Design

This research adopted a qualitative descriptive approach to explore the experiences of the study participants in depth. Data were collected through semi-structured interviews and analyzed using Reflexive Thematic Analysis (RTA), as outlined by Braun and Clarke [22]. To ensure a structured and transparent analysis, the study followed the recently developed Reflexive Thematic Analysis Reporting Guidelines (RTARG) [23]. These guidelines offer a comprehensive framework for reporting RTA, highlighting best practices, essential considerations, and potential challenges to avoid, reinforcing methodological rigor and transparency [24].

RTA was selected over other qualitative approaches—such as phenomenology, grounded theory, or content analysis—because it allows for the exploration of participants’ subjective experiences, meanings, and values, while acknowledging the active role of the researcher in the analytic process. Unlike grounded theory, which aims to generate a theory, or phenomenology, which focuses on the essence of lived experience, RTA is particularly suited for studies aiming to construct a rich, interpretative account of patterns of meaning across a dataset. This flexibility makes RTA appropriate for research that seeks to understand perspectives within complex educational and professional settings, such as the development of HP competencies among nursing students.

While RTA offers a flexible and reflexive approach to thematic analysis, it is important to acknowledge potential limitations. For example, the interpretative nature of RTA relies heavily on researchers’ subjectivity, which may introduce bias despite reflexive efforts. Additionally, RTA does not prescribe a fixed procedure, which can lead to variability in application across studies. Nonetheless, these challenges were addressed through rigorous adherence to the RTARG and continuous reflective practice, as employed in this study.

This analytical approach aligned with the central research question: “What are the perceptions and perspectives of nursing students on HP and their role as health promoters?” RTA enabled the identification and interpretation of key themes, supporting a nuanced and contextually relevant narrative.

The study protocol was retrospectively registered on the Open Science Framework (OSF) to promote transparency and adherence to open science principles. The registration is accessible at the following link: https://doi.org/10.17605/OSF.IO/WCS2E (accessed on 10 February 2025).

### 2.2. Sampling

A purposive sampling strategy was used [25] with the following inclusion criteria:

(a) Enrollment in an accredited nursing degree program.
(b) Adequate academic progression: participants were required to be in at least their second year of study and to have undertaken a minimum of one month of clinical placement, ensuring sufficient exposure to HP concepts.
(c) Willingness to participate and provide informed consent.
(d) Proficiency in the study language, ensuring the ability to provide clear, meaningful, and coherent responses.

Specifically, enrolled nursing students had to be able to express their own experience and willing to share personal insights [26]. Participants were recruited via email sent to their university-assigned mailboxes, outlining the study’s purpose and methodology. Those who expressed interest received a Google^®^ form to provide informed consent for participation and video recording.

The final number of participants was determined based on the principle of data saturation, i.e., the point at which no new relevant information or themes emerged from the data [22]. Saturation was monitored iteratively throughout the data process. After each group of interviews, the research team conducted preliminary reviews and jointly assessed whether new insights were still emerging. Saturation was deemed reached after the 17th interview; however, two additional interviews were conducted to ensure thematic redundancy and confirm saturation [22].

### 2.3. Data Collection

Data were gathered through semi-structured interviews, a widely used qualitative method that enables the collection of open-ended responses, allowing for an in-depth exploration of participants’ thoughts, emotions, and beliefs, including personal or sensitive issues. A research nurse with extensive expertise in qualitative methodologies and interview techniques conducted these in-depth interviews between December 2023 and January 2024. Three additional researchers acted as observers, taking field notes during and after each session. Interviews were conducted either in person or remotely via Microsoft Teams^®^, depending on participants’ preferences. All sessions were audio-recorded and transcribed verbatim with participants’ consent. To ensure confidentiality, transcripts were anonymized with sequential numerical codes, in accordance with the General Data Protection Regulation (GDPR) [27].

Following Braun and Clarke’s (2021) recommendations [28], the target sample size was set between 8 and 16 participants, with the option to extend up to 22 if needed to ensure saturation [22]. A total of 19 interviews were conducted, which allowed both for reaching and confirming data saturation [22]. Interviews lasted an average of 47 min, typically ranging from 40 to 55 min. Before each interview, written informed consent was obtained from all participants. Each session began with a broad, open-ended stimulus question (e.g., “What does health promotion mean to you?”), followed by a natural, free-flowing conversation guided by open-ended and reflective prompts. To maintain structure while allowing flexibility, the research team developed an interview guide with thematic areas for deeper exploration [29].

The semi-structured interview guide included open-ended questions covering nursing students’ understanding of HP, their educational and clinical experiences, perceived barriers and facilitators, and suggestions for curriculum improvement. For transparency and reproducibility, the complete interview guide is available in Appendix A. Probing questions such as “Can you elaborate?” and “What do you mean by that?” were used to elicit detailed responses. The interviewer maintained a neutral, empathetic stance and employed active listening to foster participant comfort. Key concepts and emerging themes were documented during interviews. Socio-demographic data—including age, gender, academic year, and number of clinical placements—were also collected.

### 2.4. Data Analysis

Anonymized verbatim interview transcripts were analyzed using Reflexive Thematic Analysis (RTA) according to Braun and Clarke’s six-step framework [30,31]: (a) familiarization with the data; (b) systematic coding; (c) identification of initial themes; (d) refinement and review of themes; (e) finalization and naming of themes; and (f) writing up the results. Unlike purely descriptive approaches, RTA emphasizes the interpretive role of researchers, recognizing the interconnectedness of language and experience. Codes were allowed to evolve throughout analysis [30].

After thoroughly reviewing the transcripts, two researchers independently performed a manual, line-by-line coding process, assigning concise labels to meaningful segments. The individually coded data sets were then consolidated, and an initial thematic structure was developed. Discrepancies in coding were resolved through a consensus-building process, involving reflective discussion between the two coders. When necessary, a third senior researcher was consulted to reach agreement and ensure coherence and rigor in theme development. Researchers systematically reviewed these themes to ensure they accurately captured the overarching patterns in participants’ responses. Subsequently, themes and subthemes were clearly defined, and representative quotations were selected to illustrate each theme. As RTA is interpretative, quantitative metrics such as frequency counts or inter-coder reliability were not reported, in line with its epistemological stance [22].

### 2.5. Rigor

To ensure methodological rigor and trustworthiness, the research adhered to credibility, transferability, and dependability [32]. Several strategies were implemented to enhance data fidelity and analytical accuracy. First, a complete transcription of all recorded interviews ensured transparency and consistency [32]. Second, the bracketing technique was applied, requiring researchers to document their preconceptions about the study topic to mitigate bias and strengthen the validity of findings [33]. Third, an independent analysis was conducted by a nursing-trained researcher, who was not involved in data collection and carried out initial coding independently to ensure analytical distance. This researcher then compared the coded data with those of the main analysts, contributing to the triangulation of perspectives and enriching theme development. The coding and thematic structure were subsequently verified by a qualitative research expert with prior experience in phenomenological studies. Fourth, emerging themes were collectively discussed within the research team to establish a standardized interpretation. In cases of disagreement, significant data units were revisited to refine interpretations. Disagreements in theme identification were resolved through collaborative discussions, where differing viewpoints were debated and reconciled by revisiting the data until consensus was reached. Lastly, the member-checking technique was utilized, involving a purposive subsample of five participants who had expressed a willingness for further contact. These participants were sent a summary of the preliminary themes via email and were invited to provide feedback regarding the clarity, relevance, and resonance of the interpretations with their lived experience. Their responses supported the thematic structure without requesting substantial changes, thus enhancing the credibility of the findings [34].

### 2.6. Ethical Considerations

This study adhered to international ethical guidelines for research involving human participants, ensuring the protection of their rights, privacy, and autonomy. Ethical approval was obtained from the University Ethics Committee prior to commencing data collection. Ethical acceptability was assessed according to seven fundamental criteria: scientific or social value, methodological validity, fair participant selection, favorable risk-benefit ratio, independent ethical review, informed consent, and respect for participants’ autonomy.

To recruit participants, an email invitation was sent to nursing students via their institutional email accounts. This method was chosen for its inclusivity and efficiency in reaching all eligible students, given that not all actively engage with the university’s virtual learning platform. Participation was entirely voluntary, with students informed that they could withdraw at any time without any consequences and that neither participation nor refusal would affect their academic standing. Importantly, the principal investigator had no teaching or evaluative relationship with the students at the time of recruitment, thus mitigating any risk of perceived coercion.

Prior to participation, students received detailed study information via Google Forms and provided informed consent electronically, including consent for video recording. Anonymity and data protection were rigorously ensured in accordance with the General Data Protection Regulation (GDPR, Regulation EU No. 2016/679). Each participant and interviewer was assigned a unique numerical code. Interviews were anonymized before analysis, and all communication, appointments, and data handling procedures were conducted securely.

Audio recordings and transcripts were stored on encrypted, password-protected institutional servers with restricted access only to authorized research team members. Backup copies of all data were maintained on encrypted external drives, physically secured in locked cabinets within university premises. A comprehensive data management plan guided the secure handling, storage, and eventual destruction of data in line with institutional policies. Access to raw data was logged and monitored to ensure confidentiality. Audio recordings and transcripts were stored on password-protected devices accessible only to the lead interviewer until the anonymization process was complete. The study was designed to minimize participant risk and ensure safety throughout the entire research process.

## 3. Results

The sample consisted of 19 nursing students enrolled in the 1st, 2nd, and 3rd years of their program, aged between 19 and 40 years old (mean = 23.9; SD = 5.1). At the time of the interview, 26.5% were in their first year, 26.5% in the second, and 47% in the third. The majority of participants were female (63%). Nine students had completed three or more clinical placements, six had participated in only one, and four had not yet undertaken any. Socio-demographic and curricular characteristics of the sample are presented in Table 1.

The thematic analysis of approximately 380 relevant excerpts from the interviews led to the identification of five principal themes:

1. Training Needs of Future Health Promoters;
2. Navigating Time Constraints in Health Promotion;
3. Nurses’ Awareness of Their Role in Health Promotion;
4. Perceived Need to Adopt Healthier Lifestyles;
5. Challenges of Lifestyle Management.

Each theme encompassed a range of perceived facilitators, barriers, and potential solutions, as detailed in Table 2.

### 3.1. Theme 1: Training Needs of Future Health Promoters

This theme captures students’ perspectives on their academic and clinical preparation in HP. Three overarching categories were identified: barriers, facilitators, and proposed solutions. Within these, five sub-themes emerged: feelings of inexperience and insecurity; lack of a structured training pathway (barriers); presence of knowledgeable mentors (facilitator); and the importance of practicing in a protected environment and formally recognizing HP as a learning objective (solutions).

Students frequently expressed a sense of inadequacy in HP competencies, especially in comparison to other clinical areas. This was particularly evident among those with limited internship exposure: “*In that field, I would feel less secure than in the rest… let’s say one of the fields in which I feel least secure in treating the patient*” (S17). Regarding the lack of an educational pathway, students criticized their academic preparation in HP, identifying a gap in both theoretical and practical training. One participant highlighted this issue, stating: “*I want to be a nurse, and in my opinion, health promotion is fundamental. We are the ones who work closely with patients; we have to help them promote health. But I have never had anything explained to me about it. We have barely touched on the topic, yet I think it is essential, especially now that we are at the end of our three-year program*” (S13).

Conversely, another student emphasized that positive mentoring experiences were identified as essential for learning: “*You certainly need solid background knowledge because when you engage in health promotion, you must be well-informed to convey accurate information*” (S12). To address these challenges, students proposed that HP should be explicitly integrated into the nursing curriculum and emphasized as a core learning objective. They also suggested the use of simulation-based learning: “*At the university level, more space should be given to simulations on educational topics, so that we are better prepared when we go on placement*” (S4).

### 3.2. Theme 2: Navigating Time Constraints in Health Promotion

This theme reflects students’ recognition of time limitations as a key obstacle to implementing HP in routine nursing care. Two sub-themes emerged as barriers: “the constant rush” and “conflicting priorities”, while a third sub-theme—“identifying the right moment”—was seen as a potential facilitator.

Several students described the clinical environment as characterized by a relentless pace, which often led to HP being deprioritized. As one student explained: “*The constant rush, the pressure of managing numerous tasks within a short time, forces health professionals to prioritize, often neglecting prevention and health promotion*” (S8). Others emphasized that while time constraints are a reality, experienced nurses can still find opportunities to engage in HP by being attentive and proactive. According to one participant, “*In my opinion, it’s a negotiation that starts from observing and listening*” (S3). Another student emphasized the importance of finding appropriate moments during practice to educate patients: “*It’s important to find the right time and place*” (S4). These reflections suggest the need for strategies focused on time management and clinical awareness, allowing HP to be integrated more intentionally despite workload pressures.

### 3.3. Theme 3: Nurses’ Awareness of Their Role in Health Promotion

This theme explores students’ perceptions of practicing nurses’ awareness of their responsibilities in HP, based on internship observations. Two sub-themes represented barriers—“habitual practice” and “incomplete pathways”—while “building trust” emerged as a facilitator and “professional willingness” as a potential solution. Students perceived that some nurses adhered to routine practices without integrating HP. As one student reflected: “*I don’t think health professionals believe it’s unimportant, but it’s a case of ‘this is how it’s always been done’*” (S11). Another participant pointed out that patients often received care without sufficient health-related information: “*Many procedures are performed without explanation, but patients need to understand how to take care of themselves once they go home*” (S16). In contrast, students highlighted that trust and relational continuity were key enablers of effective HP: “*You need to establish a relationship with the patient before thinking about health promotion*” (S17). To overcome these barriers, students emphasized the importance of professional motivation and intentional practice change. One student noted: “*Many things are taken for granted, but just a small effort in promotion—sometimes even unconsciously—can be helpful*” (S14). Another student summarized the issue as a matter of professional willingness: “*The main obstacle is a lack of will. And think how easy that is to solve*” (S3). This underscores that enhancing nurses’ motivation and reflective practice could serve as powerful levers for improving HP implementation.

### 3.4. Theme 4: Perceived Need to Adopt Healthier Lifestyles

This theme centers on students’ views regarding public attitudes toward health, with a focus on psychological and social barriers to adopting healthier behaviors. Two hindering sub-themes emerged: “perceived invulnerability” and “lack of resources or skills”, while “receptiveness to listening” was identified as a facilitating condition.

Participants observed that denial or underestimation of health risks was common among the public. One student described this tendency: “*It’s human nature to think, ‘it will never happen to me’*” (S2). They also recognized that not everyone has the capacity or means to maintain healthy behaviors: “*Not everyone can take care of their health*” (S17). However, students emphasized that building a trusting relationship over time could serve as a strategy to enhance patient engagement in HP. As one student explained: “*Health promotion requires continuity. It’s not a one-time intervention but an ongoing process that builds trust over time*” (S9).

### 3.5. Theme 5: Challenges of Lifestyle Management

The final theme addresses students’ reflections on their future role as health promoters, particularly in helping patients adopt sustainable lifestyle changes. Three sub-themes were identified as strategies or solutions: “taking charge of patients’ lifestyles”, “effective communication”, and “involving the family”. Students emphasized a personalized approach to care. As one participant explained: “*Every patient has a story. Once I understand them, I can tailor health promotion accordingly*” (S19). Initial patient encounters were described as critical opportunities for assessing readiness and implementing HP interventions: “*In my opinion, in taking charge, it is important to get to know the patient,* i.e., *to get to know him in the round to understand whether he is a patient who knows what health promotion is and implements these strategies or not and then to intervene there, to implement interventions*” (S11).

Students proposed that effective communication should be strengthened through specific training and tools, as it forms the foundation of any HP relationship: “*I think this is the part that should be focused on more and strategies should be elaborated so that the patient can receive more effectively the message that you want to send him/her*” (S7). They also viewed family involvement as a valuable strategy to support patient adherence to healthier behaviors. As stated by two students: “*We need to work with the entire support system—caregivers, family, friends—because the more collaborative the approach, the more effective it is*” (S12); “*Family involvement is crucial in supporting patients on their health journey*” (S4).

### 3.6. Summary of Theme Interconnections

A cross-theme analysis reveals how the identified themes are interrelated and contribute to a broader understanding of the barriers and facilitators to HP in nursing education and practice. For instance, the lack of formal training and clinical preparation (Theme 1) appears to contribute directly to students’ difficulty in integrating HP into everyday care activities (Theme 2). Similarly, limited awareness of HP among practicing nurses (Theme 3) may reinforce students’ insecurity and hinder their ability to envision themselves as competent health promoters. Moreover, challenges related to patient engagement and public attitudes toward health (Theme 4) further complicate the implementation of HP strategies. Finally, students’ reflections on their own responsibilities and the need for effective communication and family involvement (Theme 5) suggest that developing competence in HP requires both individual readiness and systemic support. Together, these themes form a complex picture of how personal, educational, professional, and contextual factors interact to shape students’ experiences with HP.

## 4. Discussion

This study aimed to explore nursing students’ perspectives on HP and their role as future health promoters. The thematic analysis revealed five main themes, focusing on training needs, time constraints, professional awareness, perceptions of healthy lifestyles, and challenges in lifestyle management. The findings highlight the need for structured interventions to enhance nurses’ role in HP, both in HP education and in clinical practice. Importantly, this study offers an original contribution by adopting a qualitative design to explore a topic that has been largely addressed through quantitative methods. By giving voice directly to nursing students, it provides in-depth insights into their lived experiences, beliefs, and perceived barriers to promoting health—perspectives that are often underrepresented in the existing literature.

Notably, this study offers novel insights into how nursing students perceive their preparedness and the contextual challenges they face in integrating HP into their future professional practice. Engaging nursing students could serve as a pivotal strategy for fostering a health-oriented culture first within the healthcare workforce and subsequently among the populations they care for. Within the findings, facilitating, hindering, and solving factors emerged and were identified based on nursing students’ perceptions on their role as a health promoter. Our results therefore contribute to the field by highlighting the critical perspective of future nurses regarding HP education and implementation—an area often explored from the viewpoint of educators or practicing professionals. Through qualitative thematic analysis, this research uncovers nuanced perceptions and concrete recommendations that can inform curricular innovation and institutional policies aimed at strengthening HP within nursing training.

One key facilitating factor identified across the various themes was the role of mentorship. Students reported that mentoring significantly enhanced their understanding and application of HP practices. This perception not only confirms previous research showing the benefits of structured mentoring programs in developing preventive care skills [35], but also highlights students’ awareness of mentorship as a key enabler of HP integration into clinical learning. Additionally, experiential learning approaches, such as simulation-based training, were seen as powerful tools in bridging the gap between theoretical knowledge and practical application. This aligns with the existing literature, while adding the important perspective that students value these methods as essential to developing confidence in HP delivery [13,36].

Another facilitating factor was the growing professional awareness of HP among students. While some nurses did not prioritize HP, students increasingly recognized its importance within the role. This supports existing evidence that embedding HP into nursing curricula strengthens professional identity around prevention [37], and our findings suggest that students internalize this value early in their training, despite observing inconsistent practices in clinical settings.

The major barrier to effective HP was time constraints, consistently reported across all themes. Students noted that the fast-paced nature of healthcare environments often forces nurses to prioritize urgent, curative tasks, leaving little room for preventive health measures. This confirms previous studies, such as that by Shan et al. (2023), which showed that high mental workload due to workflow interruptions limits HP delivery [38]. Our study advances this understanding by showing that students recognize these systemic barriers even before entering professional practice, indicating early socialization into a task-focused care model. Moreover, students observed that even when time was available, it was often used ineffectively, as nurses were unsure about when and how to incorporate HP into their routines. This corroborates findings by Melariri et al. (2022), emphasizing the persistent gap between HP knowledge and its application in clinical practice [10]. Our results suggest this gap is visible and problematic even to novice practitioners.

Another hindering factor identified was the perceived lack of preparedness among students, particularly regarding lifestyle management and encouraging healthier behaviors by patients. Nurses face challenges in motivating patients to adopt healthier habits, especially when they feel undertrained in facilitating lifestyle changes. This observation confirms Keele’s (2019) findings, which describe a lack of confidence in promoting healthy lifestyles despite theoretical knowledge [39]. Our study adds that students already anticipate this difficulty and associate it with insufficient training opportunities.

Despite these barriers, students suggested enhancing nursing curricula with targeted training in time management and preventive care delivery. Such training can equip students to identify opportunities for HP even in busy clinical settings. This supports prior evidence from Farokhzadian et al. (2020), who found that time management training improves care quality, and expands on it by showing that students themselves recognize this as a solution for integrating HP more effectively [40].

Furthermore, students emphasized the importance of training in communication skills to effectively engage patients in lifestyle discussions. Improved communication skills can enhance nurses’ ability to motivate patients toward healthier behaviors. This aligns with the established literature on patient education, but our findings suggest that students feel such skills are still underemphasized during training. Students also advocated for greater institutional support to elevate the visibility of HP within the nursing profession. This echoes calls in the literature for systemic and policy-level change [15], and our data show that future professionals are aware of the need for a cultural shift that places HP at the center of nursing practice [15].

Despite its valuable insights, this study has some limitations. The sample was limited to students from a single educational context, which restricts the generalizability of the findings. Additionally, since the data are based on self-reported perceptions, it may be influenced by students’ personal experiences and biases. Moreover, the purposive sampling strategy included primarily second- and third-year students who had completed clinical placements to ensure sufficient exposure to HP concepts. While this approach allowed us to gather informed perspectives, it inherently limits the representativeness of students at different stages of their education. We acknowledge that students’ perceptions may evolve significantly throughout their academic journey, and, therefore, more focused studies on specific year groups, as well as longitudinal research examining changes over time, are needed. These aspects have been added as important considerations for future research directions.

Future research should explore diverse educational settings and adopt longitudinal designs to assess the long-term impact of HP training on nursing students’ competencies. Furthermore, examining students’ clinical performance in HP could provide a more accurate understanding of how theoretical knowledge is translated into real-world practice. Finally, a potential limitation of the study concerns the recruitment method, which involved contacting students directly via institutional email. Although this approach was approved by the Ethics Committee and measures were taken to ensure voluntary participation and minimize perceived coercion, it may still raise concerns about undue influence, particularly in contexts where hierarchical relationships between faculty and students are more pronounced. Future studies might consider using neutral recruitment channels, such as announcements on virtual learning platforms, to further reduce any perceived pressure to participate.

Future studies should also involve the perspectives of clinical mentors and academic tutors, who play a crucial role in shaping students’ understanding and implementation of HP. Exploring how these educators perceive HP integration into nursing curricula and the challenges they face in supporting students would provide valuable insights into the barriers and facilitators of effective HP education. This could help inform targeted strategies to improve mentorship and support systems for both students and educators. In particular, mixed-methods studies could be designed to explore the impact of mentorship on students’ HP engagement, combining quantitative surveys with qualitative interviews to capture both prevalence and depth of experience.

Finally, investigating innovative teaching methods, such as experiential learning and simulation-based training, will help identify the most effective approaches for preparing nursing students to engage in HP, ultimately improving healthcare delivery and promoting better patient outcomes. We suggest conducting quasi-experimental or randomized controlled studies to evaluate the effectiveness of simulation-based activities in improving students’ confidence, communication skills, and actual implementation of HP in clinical practice. Moreover, longitudinal studies could be conducted to examine how HP-related competencies evolve across the years of nursing education and into the early stages of professional practice. Such studies could help track the long-term impact of curricular interventions and identify critical periods for reinforcing HP education.

Our findings highlight the critical need for nursing education to better integrate HP into curricula through specific, structured measures. Nursing programs should prioritize HP as a core competency by embedding it across both theoretical and clinical modules. One concrete recommendation is to include simulation-based activities focused on communication and behavioral change strategies, enabling students to practice HP interventions in realistic scenarios. Pre/post-curriculum evaluation studies could be designed to assess the impact of these changes on students’ HP knowledge, attitudes, and perceived self-efficacy. Additionally, HP should be explicitly assessed in clinical evaluation tools and learning outcome frameworks to ensure consistent reinforcement throughout the educational pathway.

Structured mentorship programs involving trained clinical preceptors should be developed to guide students in identifying and seizing HP opportunities during internships. Academic policies could also be revised to allocate protected time for HP activities during clinical placements, emphasizing HP as a legitimate component of nursing care rather than an optional add-on. In clinical practice, fostering an environment where HP is routinely integrated into care is essential. Nurses should be encouraged to develop strong communication skills, promote patient-centered care, and adopt holistic approaches to address the lifestyle factors affecting patient health. Additionally, healthcare institutions should prioritize ongoing professional development for nurses to support the continued integration of HP into their practice. These educational and policy interventions would not only strengthen nurses’ roles as health promoters but would also contribute to improved patient outcomes, greater health literacy, and the establishment of a health-oriented culture within healthcare systems.

## 5. Conclusions

This study emphasizes the need for improved HP education in nursing programs. The findings reveal gaps in both theoretical knowledge and clinical exposure, with students acknowledging the importance of HP but feeling unprepared to integrate it into practice. The use of a qualitative approach allowed us to explore students’ subjective experiences in depth, generating rich, contextualized data that quantitative surveys often fail to capture. To address these gaps, nursing curricula should prioritize HP, incorporate structured mentoring, and adopt innovative teaching methods like simulations. For example, simulation-based training can provide safe, controlled environments where students practice communication and behavioral change techniques critical to HP, while structured mentoring programs have been shown to enhance students’ confidence and practical skills in preventive care.

Educators are encouraged to embed HP-specific learning outcomes into both academic and clinical training, while policymakers should consider integrating HP competencies into national nursing education standards and accreditation frameworks. By focusing on students’ perceptions, this study offers an innovative perspective on how future nurses understand and engage with HP, highlighting the urgency of reforming educational strategies to better prepare them for this essential role. A shift in the healthcare culture is also necessary to ensure HP is a core element of nursing practice. Future research should focus on the perspectives of clinical and academic mentors to better understand how to enhance HP training and bridge the gap between theory and practice; this recommendation stems from the authors’ interpretation of students’ reported challenges and perceived gaps in mentorship during clinical training. However, it is important to acknowledge that the findings are based on a limited and context-specific sample of nursing students. As such, the results may not be generalizable to other student populations or educational settings. This limitation should be considered when interpreting the implications of the study. Embedding HP in nursing education and practice can foster a health-oriented culture, benefiting both healthcare professionals and patients.

## Figures and Tables

**Table 1:** Curricular and Sociodemographic Nursing Student’s Characteristics.

Interview Number	Age (Year)	Gender	Year of Nursing Degree Course	Enrolment Academic Year	Number of Internship Experiences
1	23	F	3	2021–2022	3
2	40	M	3	2021–2022	3
3	22	F	3	2021–2022	3
4	30	M	2	2022–2023	1
5	22	F	3	2021–2022	3
6	21	M	2	2022–2023	1
7	26	F	3	2020–2021	4
8	23	M	2	2021–2022	1
9	20	F	2	2022–2023	1
10	31	F	2	2022–2023	1
11	23	M	3	2021–2022	4
12	24	F	3	2017–2018	7
13	21	F	3	2021–2022	4
14	21	F	3	2021–2022	5
15	19	F	2	2023–2024	1
16	27	M	3	2023–2024	2
17	20	F	2	2023–2024	1
18	20	M	2	2023–2024	1
19	22	F	2	2023–2024	1

**Table 2:** Themes subthemes and quotations.

Themes	Sub-Themes	Quotations
1. Training Needs of Future Health Promoters	Feelings of inexperience and insecurity (barrier)	In that field, I would feel less secure than in the rest… (S17)
	Lack of a structured training pathway (barrier)	I want to be a nurse… We have barely touched on the topic… (S13)
	Presence of knowledgeable mentors (facilitator)	You certainly need solid background knowledge… (S12)
	Practicing in a protected environment (solution)	More space should be given to simulations on educational topics… (S4)
	Recognizing HP as a learning objective (solution)	More space should be given to simulations on educational topics… (S4)
2. Navigating Time Constraints in Health Promotion	The constant rush and Conflicting priorities (barrier)	The constant rush… forces health professionals to prioritize… (S8)
	Identifying the right moment (facilitator)	In my opinion, it’s a negotiation that starts from observing… (S3); It’s important to find the right time… (S4)
3. Nurses’ Awareness of Their Role in HP	Habitual practice (barrier)	It’s a case of ‘this is how it’s always been done’ (S11)
	Incomplete pathways (barrier)	Many procedures are performed without explanation… (S16)
	Building trust (facilitator)	You need to establish a relationship with the patient… (S17)
	Professional willingness (solution)	The main obstacle is a lack of will… (S3); Just a small effort… can be helpful (S14)
4. Perceived Need to Adopt Healthier Lifestyles	Perceived invulnerability (barrier)	It’s human nature to think, ‘it will never happen to me’ (S2)
	Lack of resources or skills (barrier)	Not everyone can take care of their health (S17)
	Receptiveness to listening (facilitator)	Health promotion… is an ongoing process that builds trust… (S9)
5. Challenges of Lifestyle Management	Taking charge of patients’ lifestyles (solution)	Every patient has a story. Once I understand them… (S19); To get to know him… and then to intervene (S11)
	Effective communication (solution)	Strategies should be elaborated… so the patient can receive more effectively… (S7)
	Involving the family (solution)	We need to work with the entire support system… (S12); Family involvement is crucial… (S4)

## Data Availability

Dataset available on request from the authors.

## Public Involvement Statement

No public involvement in any aspect of this research.

## Guidelines and Standards Statement

This manuscript was drafted in accordance with the Reflexive Thematic Analysis Reporting Guidelines (RTARG) for qualitative research using reflexive thematic analysis [41].

## Appendix A. Semi-Structured Interview Guide

**Introduction and rapport building**

- Could you please introduce yourself and describe your current year of study in the nursing program?
- How would you define Health Promotion (HP) in the context of nursing?

**Experience and perception of HP education**

- How would you describe the health promotion training you have received during your nursing studies?
- Which components of the HP training have you found particularly effective or useful?
- Are there any aspects of the HP education that you believe require improvement or further development?

**Clinical practice and health promotion**

- Can you share your experiences applying health promotion principles during clinical placements?
- What challenges have you encountered when integrating HP into clinical practice?
- What factors or supports have helped you to overcome these challenges?

**Role and competencies as health promoters**

- In your view, what is the role of nurses as health promoters?
- What key competencies should nursing students develop to effectively fulfill this role?
- How well do you feel your nursing education prepares you to develop these competencies?

**Barriers and facilitators**

- What do you perceive as the main barriers to effective health promotion within nursing education and practice?
- What facilitators or resources have you experienced that support your ability to promote health?

**Suggestions for improvement**

- What changes or enhancements would you recommend to nursing curricula to better prepare students as health promoters?
- Are there innovative teaching methods or learning experiences you think would improve HP training?

**Closing**

- Is there anything else you would like to add about your experiences or perceptions of health promotion in nursing?
